# From Cure to Combat: Deployment After Lymphoma

**DOI:** 10.3390/jcm15145327

**Published:** 2026-07-08

**Authors:** Kyle Yuan, Johnathon M. Rolwes, Sarah Darmon, Matthew J. Rendo, Joshua L. Fenderson, Anirudh Dwarakanath, James K. Aden, Michael J. Morris, Christin B. DeStefano

**Affiliations:** 1 Department of Hematology/Oncology, Walter Reed National Military Medical Center, Bethesda, MD 20814, USA; christin.destefano@usuhs.edu; 2 Department of Clinical Education, Montana College of Osteopathic Medicine, Rocky Vista University, Billings, MT 59106, USA; johnathon.rolwes@mt.rvu.edu; 3 Murtha Cancer Center Research Program, Department of Surgery, Uniformed Services University of the Health Sciences, Bethesda, MD 20814, USA; 4 The Henry M. Jackson Foundation for the Advancement of Military Medicine, Inc., Bethesda, MD 20817, USA; 5 Cancer Care Clinic, Wright Patterson Medical Center, Dayton, OH 45433, USA; 6 U.S. Army Institute of Surgical Research, San Antonio, TX 78234, USA; 7 Brooke Army Medical Center, San Antonio, TX 78234, USA; 8 David Grant USAF Medical Center, Fairfield, CA 94533, USA

**Keywords:** lymphoma, deployment, active-duty, military, readiness, demographics, hematologic malignancies, veterans affairs

## Abstract

**Background**: Lymphoma is the most common hematologic malignancy diagnosed in active-duty service members (ADSMs). Service members treated for lymphoma often face uncertainty regarding their fitness for duty, which carries implications for individual careers and force readiness. In this retrospective, exploratory study we aimed to (1) determine the proportion of previously deployed ADSMs who deployed after a lymphoma diagnosis, and (2) characterize those deployments. **Methods**: Hodgkin lymphoma (HL) and non-Hodgkin lymphoma (NHL) cases diagnosed between 2001 and 2022 were identified from the Defense Health Agency Cancer Registry using International Classification of Diseases for Oncology, Third Edition histology codes. Deployment variables were obtained from the Defense Manpower Data Center. Analysis was restricted to service members who were on active duty at diagnosis and had a history of deployment; those who had never deployed were excluded. This study was exempt from full institutional review board review. **Results**: A total of 866 previously deployed ADSMs with lymphoma were identified (455 HL, 411 NHL). Of these, 119 (13.7%; 95% CI 11.6–16.2) deployed after their lymphoma diagnosis, including 86 of 455 with HL (18.9%; 95% CI 15.6–22.8) and 33 of 411 with NHL (8.0%; 95% CI 5.8–11.1). HL survivors were significantly more likely than NHL survivors to deploy after diagnosis (*p* < 0.001). The mean interval from diagnosis to deployment was 5.1 years (SD 3.7) for HL and 4.5 years (SD 2.3) for NHL. As of 2022, all 119 individuals were alive at last follow-up. **Conclusions**: Among previously deployed ADSMs with lymphoma, roughly one in six deployed after their diagnosis and treatment, typically within five years. These findings indicate that deployment after lymphoma treatment is achievable for a significant subset of service members, highlighting both the success of cancer care in military treatment facilities and the favorable impact of modern oncologic care on operational readiness.

## 1. Introduction

Every year, more than 800 active-duty service members (ADSMs) are diagnosed with cancer [1,2]. Among these, lymphoma represents the most common hematologic malignancy. Service members who have been cured of lymphoma may face uncertainty regarding their fitness for duty, stemming from institutional stigmas and discomfort among providers managing surveillance and late effects of treatment in cancer survivors [3]. This gap in knowledge directly impacts force readiness and the careers of affected individuals.

Treatment for lymphoid malignancies has undergone a dramatic transformation over the last few decades, transitioning from toxic multi-agent chemotherapy and extended radiotherapy toward highly effective, targeted regimens utilizing novel immunotherapeutics [4,5,6]. As a result, more patients are being cured upfront and experience fewer therapy-related toxicities and late effects. Therefore, modern cancer care for lymphoma should reflect favorable benefit and risk profiles for subsequent deployment. With current efforts focusing on recruiting and retaining healthy ADSMs [7], it is conceivable that return to duty after a cancer diagnosis, such as lymphoma, is of even greater importance, particularly as operations in the Middle East and Asia-Pacific become more geopolitically complex.

While prior studies have described lymphoma subtypes and survival outcomes in the military population [8], data on post-diagnosis operational readiness are largely absent from the medical literature. As such, we performed this retrospective, exploratory study to determine the proportion of previously deployed ADSMs who deployed after a lymphoma diagnosis and to characterize the nature of those deployments.

## 2. Materials and Methods

The Defense Health Agency (DHA) Cancer Registry was used to identify all new Hodgkin lymphoma (HL) and non-Hodgkin lymphoma (NHL) diagnoses between 2001 and 2022. Cases were defined according to International Classification of Diseases for Oncology, Third Edition (ICD-O-3) histology codes 9590/3 through 9738/3. Deployment-related variables were obtained from the Defense Manpower Data Center. The cohort comprised service members who were on active duty at the time of lymphoma diagnosis and who had at least one deployment recorded during their time in service. As 38% of military servicemembers never deploy during their time in the military, those with no deployment history were excluded from analysis [9]. Deployments were identified from contingency deployment records and were defined as any combat or non-combat overseas assignment. No minimum duration threshold was imposed.

Given the nature of the study, analyses were primarily descriptive and exploratory. Continuous variables were expressed as means, medians, standard deviations (SDs), and interquartile ranges (IQRs). Additionally, 95% confidence intervals (CIs) for key proportions were calculated by the Wilson method. HL and NHL groups were compared using Fisher’s exact test for categorical variables and Welch’s *t*-test for continuous variables. Statistical package was SAS v.9.4 (SAS Institute, Inc., Cary, NC 27513, USA). This study was exempt from full institutional review board review.

## 3. Results

There were 866 previously deployed service members with lymphoma identified in the DHA Cancer Registry (455 HL, 411 NHL). Of these, 119 (13.7%; 95% CI 11.6–16.2) deployed after their lymphoma diagnosis. The demographic and deployment characteristics of those who deployed after diagnosis are shown in Table 1. As of 2022, all 119 patients were alive at last follow-up, with median follow-up from diagnosis of 10.8 years.

Among the 455 previously deployed ADSMs with Hodgkin lymphoma, 86 (18.9%; 95% CI 15.6–22.8) deployed after diagnosis, after a mean of 5.1 years (SD 3.7; median 4.2, IQR 3.7). The mean age at diagnosis was 27.5 years (range 20–48). Most were White (86%) and male (81%). Twenty-four (28%) were in the Army, 28 (33%) in the Air Force, 18 (21%) in the Navy, and 10 (12%) in the Marine Corps. Twelve deployed to Afghanistan, six to Iraq, five to both, and 63 to other locations. The mean number of post-diagnosis deployments was two, and the mean cumulative deployed time was 1075 days. Forty-one worked in intelligence/communications, 41 in repair/engineering, 11 in healthcare, seven were pilots/aircrew, and 68 worked in another occupation; 65 (75.6%) patients had more than one occupation.

Among the 411 previously deployed ADSMs with non-Hodgkin lymphoma, 33 (8.0%; 95% CI 5.8–11.1) deployed after diagnosis, after a mean of 4.5 years (SD 2.3; median 4.2, IQR 2.1). NHL histologies included diffuse large B-cell lymphoma (*n* = 18), follicular lymphoma (*n* = 8), Burkitt lymphoma (*n* = 4), and T-cell lymphoma (*n* = 3). The mean age at diagnosis was 28.9 years (range 20–55). Most were White (64%) and male (88%). Most were in the Air Force (33%), followed by Navy (30%), Army (21%), Marines (9%), and other services (6%). Seven deployed to Iraq, four to Afghanistan, two to both, and 20 to other locations. The mean number of post-diagnosis deployments was two, and the mean cumulative deployed time was 1065 days. One individual served in Infantry, one worked in intelligence/communications, six had other occupations and 25 (75.8%) had multiple occupations during their deployment.

In an exploratory comparison, HL survivors were significantly more likely than NHL survivors to deploy following their lymphoma diagnosis (18.9% vs. 8.0%; *p* < 0.001). However, the two groups did not differ significantly in the interval from diagnosis to deployment (mean 5.1 vs. 4.5 years; *p* = 0.27). Service members sustained a median deployment length of 1.1 years, and 59 (50%) deployed two or more times after diagnosis. There were generally no significant differences across HL vs. NHL demographic and deployment characteristics except a nominal difference in racial distribution (*p* = 0.02).

## 4. Discussion

In this exploratory analysis of previously deployed ADSMs with lymphoma, approximately one in six deployed again after their diagnosis. These deployments included multiple, extended, and globally diverse taskings. The finding was most pronounced for HL, with nearly one in five survivors deploying after diagnosis—significantly more often than for NHL. HL is the most common lymphoma among younger populations and generally has superior overall survival, exceeding 90% [5,6]. The improved survival and younger incidence of HL likely contributed to the higher observed deployment rate.

The roughly five-year mean interval from diagnosis to deployment aligns with community oncology practice, in which surveillance for recurrence of aggressive lymphoma generally concludes after five years. Deployment to a theater of operations is governed by DoD Instruction 6490.07 [10], which designates conditions that preclude contingency deployment without a waiver, including lymphoma. Deploying a member with such a potentially disqualifying condition requires a medical waiver, adjudicated by the geographic combatant command who weighs recurrence risk and specialty care available in theater. Accordingly, the service members in our cohort who deployed after diagnosis had either no deployment-limiting conditions or a granted waiver, supporting deployment as a formally adjudicated measure of operational readiness.

Despite advances in modern oncology, there continue to be risks of long-term health implications of lymphoma treatment. This could interfere with not only return to duty, but also general fitness and deployment restrictions. Young adult survivors of HL and NHL have historically been acknowledged as having an increased risk of cardiovascular disease, infections, long-term side effects of chemoimmunotherapy, secondary malignancies, psychosocial distress, and chronic fatigue [11], although with novel immunotherapeutics the landscape of survivorship will likely change. Given the significant percentage of ADSMs who deployed post-lymphoma, it is plausible that increased fitness, strict retention standards, and access to care may render ASDMs less prone to late effects of lymphoma treatment, although this may be reflective of the healthy-deployer effect [12].

Because patients in this study were diagnosed between 2001 and 2022, the majority were treated with conventional chemoimmunotherapy and radiotherapy. However, as modern therapeutics such as immune checkpoint inhibitors and bispecific antibodies enter routine practice, their late effects warrant future study. Autoimmune effects of nivolumab-containing regimens, such as immune thrombocytopenia, could pose issues during large-scale combat operations (LSCOs) [13,14]. While anti-CD20 bispecific antibodies are being explored in the frontline setting for B-cell NHLs, the long-term risk and effects of B-cell depletion, such as hypogammaglobulinemia and infections, are unknown [15]. There is uncertainty regarding transfusion requirements in HL survivors, where guidelines recommend lifelong blood irradiation to prevent transfusion-associated graft-versus-host disease [16,17,18], which is unfeasible when irradiated components or low-titer O whole blood are unavailable [19]. Since future LSCOs may constrain aeromedical evacuation, necessitating prolonged field care [20], future study will be critical to enable providers to manage these emerging challenges for deployed lymphoma survivors.

This study has several important limitations. First, the study design is retrospective and descriptive, limiting causal inference. There is no comparator group of lymphoma survivors who did not deploy after diagnosis; such a group could not be assembled from the available registry and deployment data. Key clinical variables, such as treatment regimen, response, relapse, performance status, and treatment-related toxicities, were also not available. These limitations preclude any meaningful assessment of differences in disease severity, treatment intensity, or fitness-for-duty patterns between deployers and non-deployers. Comparator-based studies are a key direction for future studies; they would provide researchers with the ability to identify factors associated with a successful return to deployment.

Additionally, this cohort is highly selective. Because inclusion required a history of deployment, the sample is subject to a “healthy-deployer effect” that inherently favors fitter, more deployable individuals [12]. Generally, military retention standards compound this selection, meaning survival and deployment outcomes reported here are likely more favorable than in the broader survivor population. Furthermore, the finding that all individuals who deployed post-diagnosis remained alive introduces immortal-time and survivor biases, as surviving long enough to deploy was a prerequisite for inclusion. The cohort consists entirely of United States military personnel, a population that differs substantially from civilian survivors, veterans, and international military forces. U.S. ASDMs have been shown to have both lower incidence and mortality from lymphoma compared to the general U.S. population, consistent with their generally healthier baseline, strict fitness mandates, and universal access to care [8,21].

A major recurring contributor to these limitations is the absence of integrated career and clinical data within existing registry and electronic-health-record systems. Investing in improved data stewardship regarding treatment details, fitness-for-duty determinations, medical separations, and deployment outcomes can be critical. This would enable comparator-based, multivariable, and time-to-event analyses, better inform commanders, and shape future force-management policy.

## 5. Conclusions

We demonstrate that a lymphoma diagnosis did not preclude deployment for a meaningful subset of previously deployed service members, who often deployed multiple times within several years of diagnosis with favorable survival. These findings highlight the success of cancer care delivered within U.S. military treatment facilities in contributing to operational readiness following a life-changing hematologic malignancy. Future comparator-based and clinically detailed studies are needed to identify factors that predict a successful return to deployment.

## Figures and Tables

**Table 1:** Characteristics of ADSMs who deployed following HL or NHL diagnosis.

	All (N = 119)	HL (N = 86)	NHL (N = 33)
Age at diagnosis (years)	27.9 ± 7.0	27.5 ± 6.5	28.9 ± 8.6
Sex, *n* (%)			
Men	99 (83.2)	70 (81.4)	29 (87.9)
Women	20 (16.8)	16 (18.6)	4 (12.1)
Race, *n* (%)			
White	95 (79.8)	74 (86.0)	21 (63.6)
Black	13 (10.9)	6 (7.0)	7 (21.2)
Other	11 (9.2)	6 (7.0)	5 (15.2)
Ethnicity, *n* (%)			
Non-Hispanic	99 (83.2)	74 (86.0)	25 (75.8)
Hispanic	5 (4.2)	4 (4.7)	1 (3.0)
Unknown	15 (12.6)	8 (9.3)	7 (21.2)
Stage, *n* (%)			
I	17 (14.3)	9 (10.5)	8 (24.2)
II	42 (35.3)	36 (41.9)	6 (18.2)
III	23 (19.3)	17 (19.8)	6 (18.2)
IV	9 (7.6)	6 (7.0)	3 (9.1)
Unknown	28 (23.5)	18 (20.9)	10 (30.3)
Branch of Service, *n* (%)			
Army	31 (26.1)	24 (27.9)	7 (21.2)
Air Force	39 (32.8)	28 (32.6)	11 (33.3)
Navy	28 (23.5)	18 (20.9)	10 (30.3)
Marines	13 (10.9)	10 (11.6)	3 (9.1)
Other/Unknown	8 (6.7)	6 (7.0)	2 (6.1)
Deployment location, *n* (%)			
Iraq	13 (10.9)	6 (7.0)	7 (21.2)
Afghanistan	16 (13.4)	12 (14.0)	4 (12.1)
Iraq and Afghanistan	7 (5.9)	5 (5.8)	2 (6.1)
Other	83 (69.7)	63 (73.3)	20 (60.6)
Number of deployments, *n* (%)			
1	60 (50.4)	45 (52.3)	15 (45.5)
2	26 (21.8)	21 (24.4)	5 (15.2)
3+	33 (27.7)	20 (23.3)	13 (39.4)
Deployment length, *n* (%)			
<6 months	37 (31.1)	26 (30.2)	11 (33.3)
6–<12 months	22 (18.5)	20 (23.3)	2 (6.1)
12–<24 months	22 (18.5)	15 (17.4)	7 (21.2)
24+ months	38 (31.9)	25 (29.1)	13 (39.4)
Locations per deployment, *n* (%)			
1–2	58 (48.7)	46 (53.5)	12 (36.4)
3–5	35 (29.4)	24 (27.9)	11 (33.3)
6+	26 (21.8)	16 (18.6)	10 (30.3)
Occupation, *n* (%)			
Infantry Battle	1 (0.8)	0 (0)	1 (3.0)
Engineering/Maintenance	6 (5.0)	6 (7.0)	0 (0)
Communication/Intelligence	7 (5.9)	6 (7.0)	1 (3.0)
Healthcare	2 (1.7)	2 (2.3)	0 (0)
Other	13 (10.9)	7 (8.1)	6 (18.2)
Multiple Occupations	90 (75.6)	65 (75.6)	25 (75.8)

Characteristics of ADSMs who deployed following HL or NHL diagnosis. Exploratory HL versus NHL comparisons (Fisher’s exact test for categorical variables; Welch’s *t*-test for continuous variables): age at diagnosis *p* = 0.42; sex *p* = 0.58; race *p* = 0.02; ethnicity *p* = 0.20; branch of service *p* = 0.85; deployment location *p* = 0.18; number of deployments *p* = 0.20; deployment length *p* = 0.16; locations per deployment *p* = 0.21; stage (I–IV) *p* = 0.06.

## Data Availability

The original contributions presented in this study are included in the article. Further inquiries can be directed to the corresponding author.

## Abbreviations

The following abbreviations are used in this manuscript:

ADSM	Active-duty service member.
HL	Hodgkin lymphoma.
NHL	Non-Hodgkin lymphoma.
DHA	Defense Health Agency.
SD	Standard deviation.
LSCO	Large-scale combat operations.
